# Influence of implant diameter on implant survival rate and clinical outcomes in the posterior area: a systematic review and meta-analysis

**DOI:** 10.1186/s12903-023-02962-8

**Published:** 2023-04-21

**Authors:** Paolo Pesce, Massimo Del Fabbro, Laura Modenese, Stefano Sandron, Luca Francetti, Gaetano Isola, Luigi Canullo, Maria Menini

**Affiliations:** 1 Department of Surgical Sciences and Integrated Diagnostics (DISC), University of Genoa, Ospedale S. Martino, L. Rosanna Benzi 10, 16132 Genoa, Italy; 2 Department of Biomedical, Surgical and Dental Sciences, University of Milan, Milan, Italy; 3 Fondazione IRCCS Ca’ Granda Ospedale Maggiore Policlinico, Milan, Italy; 4 Department of Biomedical, Surgical and Dental Sciences, University of Milan, Milan, Italy; 5 IRCCS Orthopedic Institute Galeazzi, Dental Clinic, Milan, Italy; 6 Department of General Surgery and Surgical-Medical SpecialtiesSchool of Dentistry, University of Catania Via S, Sofia 78, Pad. 2 Piano -1 Stanza 53, 95124 Catania, Italy

**Keywords:** Dental implants, Narrow implants, Bone resorption, Implant diameter, Systematic review, Meta-analysis

## Abstract

**Objective:**

The aim of the present systematic review was to test the hypothesis that the diameter of implants inserted in the posterior area affects implant survival rate, prosthetic survival rate and peri-implant parameters (bleeding on probing (BoP), marginal bone loss (MBL), pocket probing depth (PPD)).

**Materials and methods:**

An electronic search of studies published until December 2021 was done on three databases (Pubmed, Scopus, Cochrane) independently by two authors. Clinical trials comparing implant survival rate, BoP, MBL and PPD among narrow diameter implants (NDI: ≥ 3.0 mm to < 3.75 mm) and regular diameter implants (RDI ≥ 3.75 mm to < 5 mm) were included. Data were independently extracted by two reviewers. Risk of bias was evaluated according to the *Cochrane* risk-of-bias *tool* for randomized studies and to the *Joanna Briggs Institute* Critical Appraisal tools for non-randomized ones. A pair-wise meta-analysis was conducted on the included studies.

**Results:**

Seven articles were included out of the 4291 identified from the digital research. Overall, a total of 939 implants were inserted (319 NDI, 620 RDI). Only one study was judged at serious risk of bias. No statistically significant difference was found in implant survival rate (risk ratio 1.01 (95% CI [0.98 to 1.04], *P* = 0.67)) while the difference was significant for BoP (mean difference 2.89 (95% CI [0.30 to 5.48] mm, *P* = 0.03)) with higher values for NDI. Higher MBL was identified among regular diameter implants (mean difference -0.15 mm (95% CI [-0.32 to 0.01 mm], *P* = 0.07). No statistically significant differences were identified for prosthetic survival and PPD.

**Conclusions:**

No differences were found in implant survival rate between narrow and regular implants. A higher BoP was identified among narrow implants, but there was no higher bone loss. It is not possible to draw definitive conclusions about the use of narrow-diameter implants in the posterior region.

**Supplementary Information:**

The online version contains supplementary material available at 10.1186/s12903-023-02962-8.

## Introduction

The success of implant rehabilitation in the posterior regions of the jaws depends on several factors such as the location and extent of the edentulous area, remaining teeth status, patient compliance with oral hygiene, patient-related factors [1, 2], including possible parafunctions, the condition of the opposing arch, implant surface characteristics [3–5] but especially by the quality, the height, and the width of residual bone [6]. In these areas, due to the intensity of the masticatory forces developed [7], the gold standard is the insertion of a regular or a wide implant to replace missing elements [8]. Following horizontal bone resorption, however, this practice is not always possible unless proceeding, before or simultaneously, with bone regeneration techniques [9].

In modern dentistry, many different augmentation procedures, depending on the location and size of the defect, have now been developed to increase bone width: expansion with osteotomes [10], autologous bone grafts [11], osteogenic distraction [12], guided bone regeneration [13] and crestal expansion techniques [14]. Although most of the histological [15] and clinical [16] aspects of these procedures are known, they are not without complications [16, 17]. The most common inconveniences of these procedures are post-operative pain, lengthening of healing time, nerve damage, bone fractures, hemorrhage, secondary infections due to wound dehiscences, and implant or augmentation failures [18, 19].

In patients with a reduced thickness of the residual alveolar crest, the use of narrow-diameter implants (NDIs) could be a plausible treatment option to overcome these drawbacks [20].

Since no consensus has been reached in the literature about classifying dental implants by their diameter, in the present meta-analysis it was decided to use the classification proposed by Al-Johany and Al Amri [21]. This classification identified four different groups of implants based on the implant diameter, defined as the width of the dental implant at the neck area:

- Extra-narrow (< 3.0 mm)
- Narrow (≥ 3.0 mm to < 3.75 mm)
- Regular (≥ 3.75 mm to < 5 mm)
- Wide (≥ 5.0 mm).

Originally, narrow implants were developed to replace dental elements with a small clinical crown or in cases where the interdental or interimplant space was reduced (upper lateral or lower incisors areas) [22]. The use of these implants in the posterior jaws was considered unfavorable due to prosthetic and biomechanical aspects. The emergence profile of posterior teeth is hardly compatible with a narrow implant neck [6] that could make oral hygiene difficult. Additionally, bite force in the posterior area can reach very high values creating high stress on abutments and implants [7]. For these reasons, more complications are expected using NDIs in posterior areas.

Consequently, the main purpose of this meta-analysis was to test the hypothesis that the diameter of implants inserted in the posterior area (premolar and molar area) affects implant survival rate, prosthetic survival rate and peri-implant parameters (bleeding on probing (BoP), marginal bone loss (MBL), pocket probing depth (PPD))**.**

## Materials and methods

The present systematic review with a meta-analysis was created following the Preferred Reporting Items for Systematic Review and Meta-Analysis (PRISMA) guidelines (http://www.prisma-statement.org/) and the protocol was registered with PROSPERO (CRD42022322379).

The focused question was: “Is there a difference in implant survival rate, prosthetic survival and periodontal parameters (bleeding on probing (BoP), marginal bone loss (MBL), pocket probing depth (PPD)) among dental implant of narrow and regular diameter inserted in posterior areas of the jaw?

The focused question was developed using the PICO scheme:

- Population: Healthy patients rehabilitated with dental implant in posterior areas.
- Intervention: Narrow diameter implants (≥ 3.0 mm to < 3.75 mm).
- Comparison: Regular diameter implants (≥ 3.75 mm to < 5 mm).
- Outcomes: Implant cumulative survival rate and/or prosthetic survival rate and/or bleeding on probing and/or probing pocket depth and/or marginal bone loss.

### Search strategy and study selection

Electronic research was carried out using three main database, PubMed (MEDLINE), Scopus and the Cochrane Central Register of Controlled Clinical Trials (CENTRAL). The last search was performed in December 2021. The investigation was performed using the following search strategy adapted for each database: (Dental implant) AND (diameter or narrow or small) AND (posterior or molar or premolar). In order not to leave out any study of interest, different Boolean combinations of the following terms was also used for the search: ‘dental implants’, ‘implant diameter’, ‘narrow diameter’, ‘small diameter’, ‘regular diameter’, ‘posterior area’, ‘posterior jaws’, ‘molar area’.

The references of all the included studies and relevant systematic reviews were screened for additional studies and no language nor date of publication restriction were adopted.

Two authors (SS and LM) reviewed the papers, screened titles, abstracts and full texts and Cohen’s Kappa was used to assess the inter-examiners agreement. In case of doubt, a third co-author (PP) was consulted. Full texts of all the eligible articles were downloaded and in case of exclusion, the reasons for exclusion were registered.

### Eligibility criteria

For the development of this meta-analysis, only studies with the following characteristics have been considered: (1) Randomized Controlled Trials (RCT), clinical trials and observational studies, both retrospective and prospective; (2) human studies with healthy patients; (3) studies with at least 1-year follow-up; (4) presence of narrow and regular implants inserted in the posterior areas of the jaws.

Instead, articles have been excluded in case of: (1) articles with duplicate reports of earlier trials; (2) articles whose full texts were unavailable; (3) case reports; (4) pilot studies; (5) animal studies; (6) in-vitro studies; (7) systematic reviews and meta-analyses; (8) studies comparing Titanium-Zirconia (Ti-Zr) and pure-Titanium implants; (9) studies comparing implants inserted in pristine bone with implants placed in augmented bone; (10) absence of primary outcomes.

No article has been excluded for the year of publication or for the language.

### Data extraction

After carefully selecting the studies to be included in this meta-analysis, two authors (SS and LM) extracted data using a Microsoft Excel spreadsheet. The following information was extracted: year and journal of publication, authors, title, type of study (RCT, prospective or retrospective) and study design (parallel or split-mouth), country in which the study was performed, presence of sponsors, presence or absence of smoking patients, age of patients, follow-up period in months, location of the implants (anterior/posterior and superior/inferior), implant surface, type of loading, prosthesis retention (screw or cemented) and prosthesis material, total number of implants inserted (narrow, regular), implant diameter in mm and outcomes. For each category of dental implants, narrow diameter (NDI) and regular diameter (RDI), the following outcomes were evaluated: Implant Survival Rate, Prosthesis Success Rate, Marginal Bone Loss (MBL), Bleeding on Probing (BoP) and Probing Depth (PPD). Since some of the selected articles did not provide all the necessary information, authors were contacted in order to obtain the missing data.

### Risk of bias assessment

Two co-authors (MDF, PP) independently assessed the articles for the risk of BIAS. RCTs were evaluated according to *Cochrane* risk-of-bias *tool* for randomized trials and analyzed on seven different BIAS. Cohort studies were evaluated using the *Joanna Briggs Institute* Critical Appraisal tools and evaluated according 10/11 different domains.

An overall judgment of Bias was finally assigned to each included article. For RCTs the possible risk was low, moderate, or high. For cohort studies and case series the possible risk was low, moderate, serious, and critical.

### Data analysis

Pairwise meta-analysis (NMA) was undertaken to obtain estimates for primary outcomes. The estimate of effect of an intervention was expressed as mean differences (MDs) or relative risk (RR), as appropriate, along with 95% confidence intervals (CIs). Heterogeneity among included studies was assessed using Cochran's test for heterogeneity, considering a significance threshold of *P* < 0.1. The software Review Manager (RevMan Version 5.4.1, The Cochrane Collaboration, 2020) was used for pairwise meta-analysis. A fixed-effects model was adopted. In case of significant heterogeneity, a random-effects model was applied and, in case of persistence of heterogeneity, studies with high risk-of bias were removed, and the analysis run again. When feasible, missing standard deviations were estimated using the methods described in the Sect. 7.7.3 of the Cochrane Handbook for Systematic Reviews of Interventions, Version 5.1.0 (Higgins 2011).

## Results

### Study selection

**Fig. 1:**
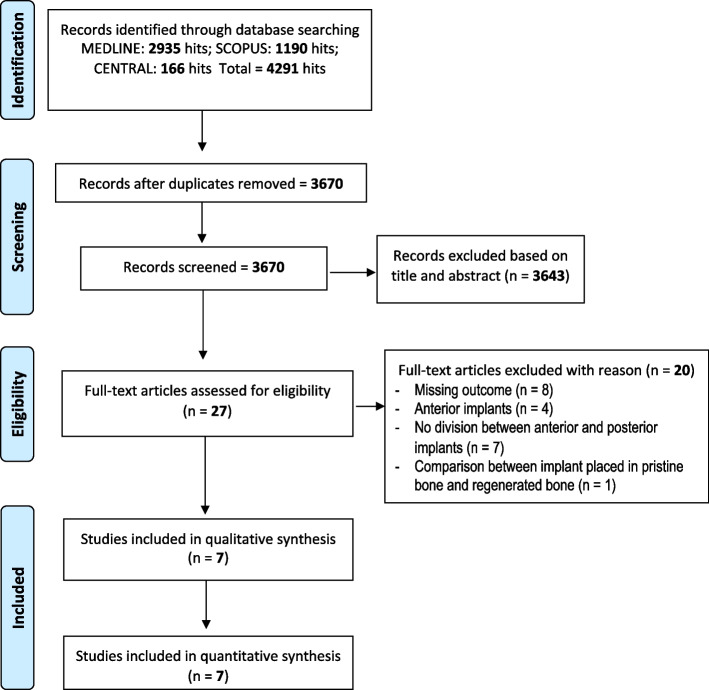
Flow-chart of the selection process

The online databases investigation (MEDLINE: *n* = 2935; CENTRAL: *n* = 166; SCOPUS: *n* = 1190) led to find 4291 relevant articles. After duplicates removal, 3670 articles were evaluated. Of these, 3643 were excluded after the screening of title or abstract because they did not fulfill the inclusion criteria. The remaining 27 articles were selected for full-text reading. The screening of full texts led to the exclusion of other 20 papers. The kappa value for inter-reviewer agreement was 0.91 indicating an almost perfect agreement (Landis and Koch scale). A total of 7 studies were included in the meta-analysis [23–29]. The flow-chart of the selection process is reported in Fig. 1.

Two of the nine authors contacted for additional information, provided the missing data and articles were included in the meta-analysis.

### Description of included studies

Of the 7 articles included in the meta-analysis, 5 were retrospective studies (RS) [23–25, 27, 28], one was an RCT [26] while the other was a prospective non-randomized trial (PS) [29].

One article by De Souza et al. [26] used a split-mouth approach, while all the remaining papers were parallel studies. All the information are reported in Tables 1, 2, and 3.

**Table 1:** Description of the included studies

No	Pub Year	Author	Type of study	Parallel (P) / Split mouth (S)	Country	Sponsor Y/N	Smokers incl. Y/N	Age (mean, SD, range)	Total implants	implants division Sup/Inf	Implant Surface	Type of loading	Cemented / Screw prosthesis	Prosthesis material	Single / Splinted	Follow-up, months
1	2020	Alrabiah et al.	RS	P	Saudi Arabia	Y	N	39.6 (31–49) N 41.4 (34–48) R	100	/ No division between anterior and posterior for this data	Moderately rough surface	Delayed loading	Both	/	Single (46 N – 52R) Splinted (68 N- 69 R)	53 (35–69) N 52 (34–70) R
2	2017	de Souza et al.	RCT	S	USA	N	Y (< 10 cigarettes)	59.2	44	9–13 N 10–12 R	Rough SLA surface	Delayed loading	Screw	/	Single	36
3	2017	Pieri et al.	RS	P	Italy	N	Y (< 20 cigarettes)	61.02 ± 9.76 N 56.77 ± 9.86 R	239	/ Data not included	/	Delayed loading	Both	Full-zirconia, zirconia– ceramic, metal-ceramic, and titanium-composite FPDs	Splinted	60
4	2019	Al-Shibani et al.	RS	P	Saudi Arabia	Y	N	41.6 (30–50)	86	/ Data not included	/	Delayed loading	/	/	NA	36
5	2003	Garlini et al.	RS	P	Italy	N	Y (< 10 cigarettes)	24–75	115	/ No division between NDI, RDI and WDI for this data	Osseotite surface	Delayed loading	Screw	Metal-ceramic (single prosthesis and FPP) and acrylic resin with a metal framework (full-arch)	Data not specified (both single both splinted)	60
6	2014	Mangano et al.	PS	P	Italy	N	Y	49.1 ± 11.5 (24–74)	101	/ No division between NDI, RDI and WDI for this data	Rough surface	Delayed loading	Cemented	Metal-ceramic	Single	120
7	2006	Romeo et al.	RS	P	Italy	N	Y (< 10 cigarettes)	55.8 (21–74)	254	27–48 N 82–97 R	Titanium-Plasma Spray (TPS) surface	Delayed loading	Both	/	Single (23 N – 50 R) Splinted (99 N – 158 R)	84

*RS* retrospective study, *RCT* randomized controlled trial, *PS* prospective study, *N* narrow-diameter implants, *R* regular-diameter implants

**Table 2:** Outcomes of narrow implants

No	Author	n. Narrow implants	Narrow diameter	Implant Survival Rate (Narrow)	Prosthesis Success Rate (Narrow)	MBL (Narrow)	BoP (Narrow) % sites	PPD (Narrow)
1	Alrabiah et al	53	3.3	/	/	1.4 ± 0.2	35.1 ± 6.4	3.3 ± 0.6
2	de Souza et al	22	3.3	100 (22/22) – > 1 year 95 (19/20) – > 3 years	95.45 (21/22) – > 1 year 90 (18/20) – > 3 years	0.49 ± 0.27 – > 1 year 0.58 ± 0.39 – > 3 years	/	/
3	Pieri et al	113	3.0	98.2 (111/113)	97.95 (48/49)	0.95 ± 0.84	/	/
4	Al-Shibani et al	47 (22D + 25ND)	3.3	/	/	0.15 (0.1–0.4)	21.3 (12.4–26.7)	2.4 (2.1–3.3)
5	Garlini et al	4	3.25	100 (4/4)	/	/	/	/
6	Mangano et al	5	3.3	100 (5/5)	100 (5/5)	/	/	/
7	Romeo et al	75	3.3	98.6 (74/75)	/	0.5 ± 0.3 – > load 1.5 ± 1.3 – > 7 years	/	2.3 ± 1.0 – > load 2.4 ± 1.1 – > 7 years

*MBL* mean bone loss, *BoP* bleeding on probing, *PPD* pocket probing depth

**Table 3:** Outcomes of regular implants

No	Author	n. Regular implants	Regular diameter	Implant Survival Rate (Regular)	Prosthesis Success Rate (Regular)	MBL (Regular)	BoP (Regular) % sites	PPD (Regular)
1	Alrabiah et al	47	4.0	/	/	1.7 ± 0.5	31.6 ± 8.8	3.2 ± 1.8
2	de Souza et al	22	4.1	100 (22/22) – > 1 year 100 (20/20) – > 3 years	100 (22/22) – > 1 year 95 (19/20) – > 3 years	0.42 ± 0.24 – > 1 year 0.53 ± 0.46 – > 3 years	/	/
3	Pieri et al	126	4.0 (77) 4.5 (49)	96.8 (122/126)	96.5 (56/58)	1.2 ± 0.86	/	/
4	Al-Shibani et al	39 (20D + 19ND)	4.1	/	/	0.2 (0.1–0.4)	18.7 (14.8–21.4)	2.4 (2.0–3.1)
5	Garlini et al	111	3.75	98.1 (109/111)	/	/	/	/
6	Mangano et al	96	4.1	97.91 (94/96)	98.9 (93/94)	/	/	/
7	Romeo et al	179	4.1	98.3 (176/179)	/	0.6 ± 0.2 – > load 1.6 ± 1.1 – > 7 years	/	2.4 ± 0.9 – > load 2.5 ± 1.2 – > 7 years

*MBL* mean bone loss, *BoP* bleeding on probing, *PPD* pocket probing depth

Most of the publications were not sponsored, only two articles were financed [23, 28]. Five studies included smoking patients while the other 2 studies excluded them [23, 28].

Regarding the age of the patients treated, 3 studies selected patients with an average age of less than 50 years [23, 28, 29], while 3 other studies selected patients with an average age between 50–65 years [24, 26, 27]. The remaining study, instead of reporting the average age of patients, provides the range (24–75 years) [25].

In total, 939 implants were inserted in the posterior areas of the jaws; of these, 319 are NDI and 620 are RDI. In all the included studies implants were inserted both in the upper both in the lower jaw. All the implants were rehabilitates following a delayed protocol and five authors provided information on the used rough implant surface [23–26, 29]. Dealing with prosthetic procedures, 2 studies [25, 26] used only screwed prostheses, one study [29] used only cemented prostheses, while three studies [23, 24, 27] used both retention systems; the remaining study [28] did not include this data. Only three studies provide information regarding the prostheses material: Pieri et al. [27] used full-zirconia, zirconia-ceramic, metal-ceramic, and titanium-composite prosthesis; Garlini et al. [25] used metal-ceramic and acrylic resin with a metal framework prosthesis; Mangano et al. [29] used only metal-ceramic prosthesis.

Implants were then followed for different periods: 2 articles had a follow-up of 3 years [26, 28], 1 study had a follow-up of 53 months [23], 2 studies had a follow-up of 5 years [25, 27], while the remaining 2 studies lasted 7 [24] and 10 [29] years respectively.

We decided to follow the Al-Johany classification [21] for the implant diameters. About NDIs, five of the included studies used 3.3 mm diameter implants [23, 24, 26, 28, 29]. The remaining studies used 3.0 mm [27] and 3.25 mm [25] diameter implants. About RDIs, four studies used 4.1 mm implants [24, 26, 28, 29], one paper used 3.75 mm implants [25] and another study used 4.0 mm implants [23]. In the remaining study [27], however, 77 implants had a diameter of 4.0 mm while the remaining 49 had a diameter of 4.5 mm.

### Risk of bias

The risk of bias among the included studies is reported in Table 4. In our evaluation the RCT [26] included was classified as a moderate risk of bias. Among the cohort studies two were classified as low risk of bias [27, 28], one as moderate risk of bias [23], and one as serious risk of bias [24]. Among the case series, one article was judged as low risk of bias [29] and one as moderate risk [25].

**Table 4.**
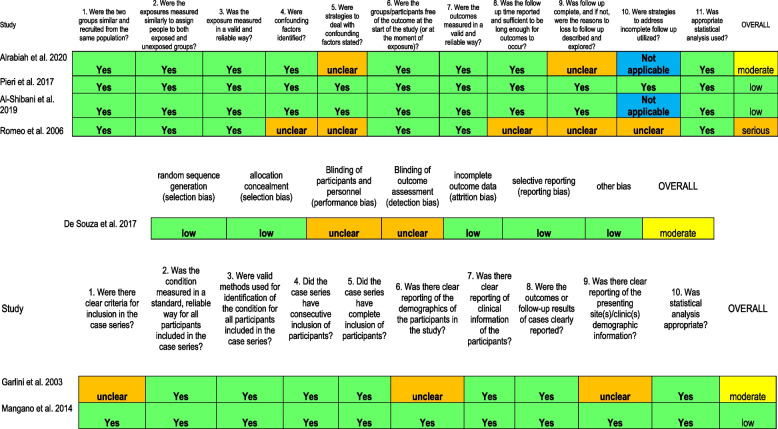
Risk of BIAS Alrabiah et al. 2020 [23], Pieri et al. 2017 [27], Al-Shibani et al. 2019 [28], Romeo et al. 2006 [24], De Souza et al. 2018 [26], Garlini et al. 2003 [25], Mangano et al. 2014 [29]

### Pair-wise meta-analysis

#### Implant survival

**Fig. 2:**
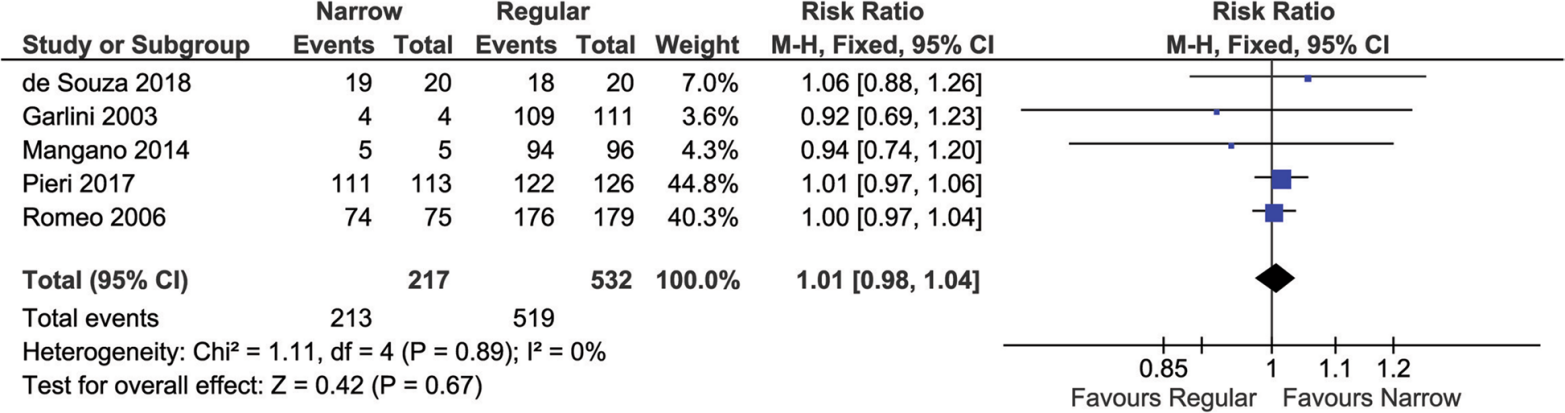
Pair-wise meta-analysis of implant survival

Implant survival rate was evaluated in five studies [24–27, 29] and is reported in Fig. 2. Overall, no significant difference in survival rate was found between narrow and regular diameter implants (1.01 (95% CI [0.98 to 1.04], *P* = 0.67)). A low heterogeneity among studies was found (I^2^ < 0.00001, *P* = 0.89).

A sub-analysis was performed on single implants (not splinted) and is reported in supplementary Fig. 1. No significant difference in survival rate was found between narrow and regular diameter implants (1.01 (95% CI [0.88 to 1.17], *P* = 0.87)). A low heterogeneity among studies was found (I^2^ = 0%, *P* = 0.45).

### Prosthetic survival

**Fig. 3:**
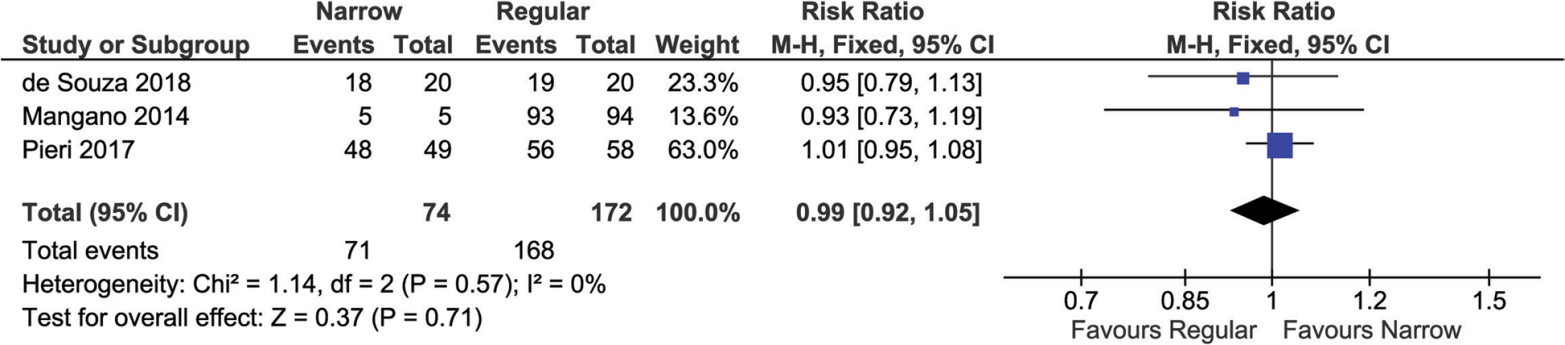
Pair-wise meta-analysis of prosthetic survival

Prosthetic survival rate was evaluated in three studies [26, 27, 29] and is reported in Fig. 3. Overall, there was not a significant difference among narrow or regular diameter implants (0.99 (95% CI [0.92 to 1.05], *P* = 0.71)). A low heterogeneity among studies was found (I^2^ < 0.00001, *P* = 0.57).

A sub-analysis was performed on single implants (not splinted) and is reported in supplementary Fig. 1. No significant difference in survival rate was found between narrow and regular diameter implants (0.94 (95% CI [0.82 to 1.09], *P* = 0.41)). A low heterogeneity among studies was found (I^2^ = 0%, *P* = 0.91).

### Bleeding on probing

**Fig. 4:**
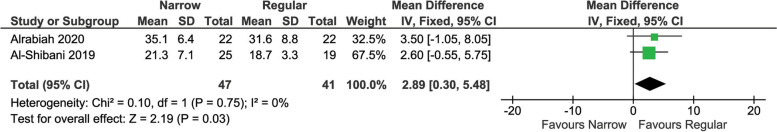
Pair-wise meta-analysis of BoP

BoP was evaluated in two studies [23, 28] and is reported in Fig. 4. Overall, there was a significant difference with higher BoP among narrow diameter implants (2.89 (95% CI [0.30 to 5.48], *P* = 0.71)). A low heterogeneity among studies was found (I^2^ < 0.00001, *P* = 0.75).

### Mean bone loss

**Fig. 5:**
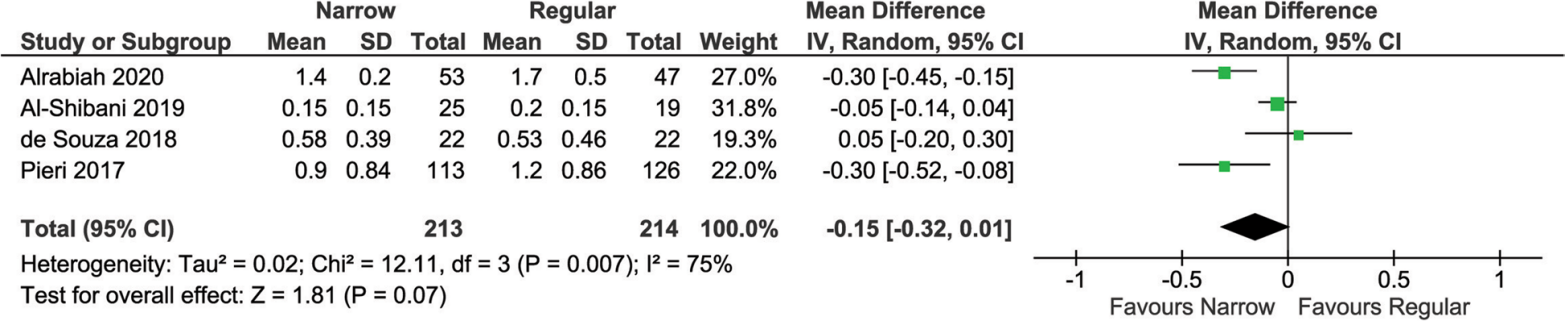
Pair-wise meta-analysis of MBL

MBL was evaluated in five studies [23, 24, 26–28]. Since there was high heterogeneity, a random effects model was used, and due to persistence of heterogeneity, the analysis was run after removing the study with a serious ROB [24] (Fig. 5). A non-significant (*P* = 0.07) difference between NDI and regular diameter implants was found, with a trend in favor of NDI implants (-0.15 mm (95% CI [-0.32 to 0.01 mm], *P* = 0.07). A significant heterogeneity among studies was found (I^2^ = 75%, *P* = 0.007).

### Probing pocket depth

**Fig. 6:**
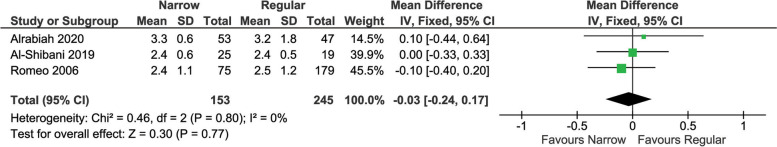
Pair-wise meta-analysis of PPD

PPD was evaluated in three studies [23, 24, 28] and is reported in Fig. 6. Overall, there was not a significant difference among regular and narrow diameter implants (-0.03 (95% CI [-0.24 to 0.17], *P* = 0.77)). A low heterogeneity among studies was found (I^2^ < 0.00001, *P* = 0.80).

## Discussion

The aim of the present meta-analysis was to assess the effect of implant diameter on clinical outcomes of implant- prosthetic rehabilitation on the posterior areas of the jaws. NDIs are generally used when interdental space and residual bone width are limited [30]. Since alveolar ridge resorption after tooth loss can result in loss of up to 50% of bucco-palatal bone volume in the first 12 months [31–33], their use allows to restore edentulous sectors avoiding regenerative surgery [34, 35].

It has been demonstrated that narrow-diameter implants (≥ 3.0 mm to < 3.75 mm) could be a predictable treatment also for posterior jaws rehabilitations [22, 24, 36], although their use for placement of single crowns in areas subjected to high magnitude forces was not recommended in the past [37] because of their reduced mechanical strength.

Results of the present pair-wise meta-analysis reveal that there are no differences between NDIs and RDIs in terms of implant survival and prosthetic survival. Considering that splinting implants together could represent a confounding factor, a sub-analysis was conducted among single rehabilitated implants. Also, in this case no statistically significant differences between NDIs and RDIs in terms of implant survival and prosthetic survival were identified. Previous reviews had already demonstrated the reliability of using narrow-diameter implants, reporting survival rates comparable to that of regular diameter implants [30, 38, 39]. Sohrabi and colleagues in their review (2012), assessing narrow dental implants to rehabilitate both anterior and posterior sectors, point out that failure rate appeared to be higher in NDIs with a length of 13 mm or less than in longer one [38]. In a recent meta-analysis González-Valls et al. (2021) [30] described the survival rate of NDIs, placed both in anterior and posterior areas, after 36 months of follow-up, at 97%. However, this result is slightly lower than that reported in a previous review, equal to 98.6%, evaluating narrow-diameter implants, during a period from 1 up to 12 years, placed only in the posterior jaw [40]. The meta-analysis of Ma and colleagues, comparing NDIs and RDIs in terms of implant and prosthesis success rate and MBL after 1 and 3 years, report similar survival rate for narrow-diameter implants (98.71%) also emphasizing that there is no significant statistical difference between the implant survival of NDIs and RDIs, after the same follow-up period [39]. However, the previously mentioned meta-analysis, included implants placed both in anterior and posterior regions, differently from the inclusion criteria we adopted. The meta-analysis by Alrabiah (2019) evaluated implants inserted only in posterior areas and did not show a significant overall difference in survival rates between narrow-diameter implants (NDIs) and regular-diameter implants (RDIs), however it demonstrated a favorable trend towards narrow-diameter dental implants [41].

It should be stressed that in load-bearing areas, particularly using NDIs, implant success is influenced not only by bone quality and occlusal forces, but also by the restoration emergence profile that must be adequate to allow the maintenance of good hygiene and soft tissue peri-implant health. This is difficult when narrow implants are used in posterior area. The larger size of the crowns could force the technician to create over-contours that are difficult to clean. Although plaque index was not evaluated in the present study, this might explain why higher BoP was found around narrow diameter dental implants compared to regular implants, although there was no evidence that implant diameter affects PPD. In contrast, regarding MBL, since there was high heterogeneity, a random effects model was used, and due to persistence of heterogeneity, the analysis was run after removing the study with a serious ROB [24]. A non-significant (*P* = 0.07) difference between NDI and regular diameter implants was found, with a trend in favor of NDI implants. A narrow diameter might help in maintaining a sufficient bone volume all around the implant, and this aspect might be more important than other factors, such as plaque accumulation, in order to prevent peri-implant bone resorption over time [42].

Other meta-analysis found similar values of marginal bone loss comparing regular and narrow diameter implants, although rehabilitations of the anterior as well as posterior areas were considered [30, 39]. It must be considered that several variables other than implant diameter might have affected the outcomes, including the available bone quantity.

With regards to prosthetic survival, the conclusions of the present analysis are in according to those of Ma and colleagues (2019) that reported no significant differences between NDIs and RDIs after 1 year and 3 years of follow-up (3-year prosthesis success rate of 89.25% and 96.55% for narrow and regular diameter implants respectively) [39].

Some limits of the present meta-analysis must be acknowledged. First of all, titanium-zirconium implants were excluded from the present review. To reduce the risk of fatigue fracture, a new titanium-zirconium alloy (TiZr; 83–87% titanium added to 13–17% zirconium) has recently been introduced for the fabrication of narrow-diameter implants [43]. The addition of zirconium seems to increase the alloy’s resistance to corrosion [44] and improve the fatigue stress resistance [45–47]. TiZr narrow implants also seems to guarantee the achievement of implant and prosthesis survival rate consistent with those obtainable with regular diameter implants [43]. Although the use of this new alloy has proven to be predictable both in the anterior and posterior sector of the jaws, in the present meta-analysis, it was decided to excluded studies evaluating Titanium-Zirconia (TiZr)—focusing only on those analyzing commercially pure titanium implants.

Another limit of the present meta-analysis, to be mentioned for the impact it may exert on the analyzed outcomes, is the heterogeneity among the included studies in terms of study design (retrospective and prospective studies), implant abutment surface [48–50], prosthetic rehabilitation type, as both single crowns and partial rehabilitation were adopted. Particularly, De Souza et al. (2018) [26] and Mangano et al. (2014) [29] placed only single crowns; Pieri et al. (2017) [27] employed partial rehabilitations, while Alrabiah et al. (2020) [23], Romeo et al. (2006) [24] and Garlini et al. (2003) [25] use both prosthetic solutions. Only Al-Shibani et al. (2019) [28] did not provide this information. In addition, Mangano et al. [29] used only short implants with few NDIs inserted. However, we decided to include it as done by Ma et al. [39] in their meta-analysis. The prosthetic rehabilitation can have a significant impact on the implant survival rate, peri-implantitis rate, and the rate of prosthesis complications. Particularly when narrow diameter implants are used clinicians may tend to splint the implant crowns to achieve better distribution of biting force to avoid excessive force on one implant. For this reason, a sub-analysis was performed and no statistically significant differences between NDIs and RDIs in terms of implant survival and prosthetic survival were identified among single rehabilitated implants.

## Conclusions

Within the limits of the present systematic review, it is not possible to draw definitive conclusion about the use of narrow-diameter implants in the posterior region. No statistically significant differences were found for implant survival rate and prosthetic survival rate. RCT are needed to clarify this topic.

## Supplementary Information

**Additional file 1.**

**Additional file 2.** 

## Data Availability

All data generated or analyzed during this study are included in this published article.
