# Supplementary figures and images for: Influence of implant diameter on implant survival rate and clinical outcomes in the posterior area: a systematic review and meta-analysis
Source: BMC Oral Health. 2023 Apr 21;23:235. doi: 10.1186/s12903-023-02962-8 (PMC10122303; doi:10.1186/s12903-023-02962-8)

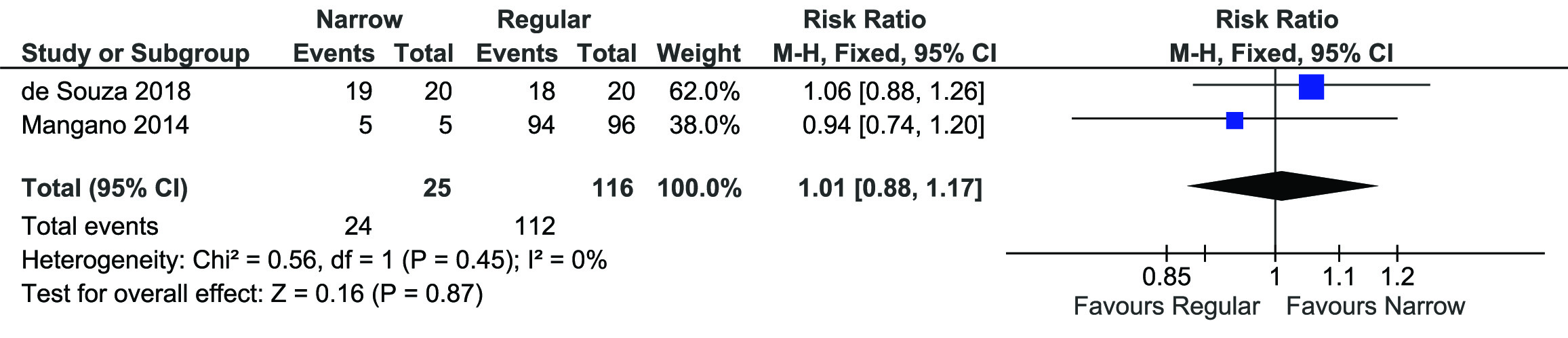

Supplement: Supplementary file 1 — Additional file 1. [file 12903_2023_2962_MOESM1_ESM.jpg]

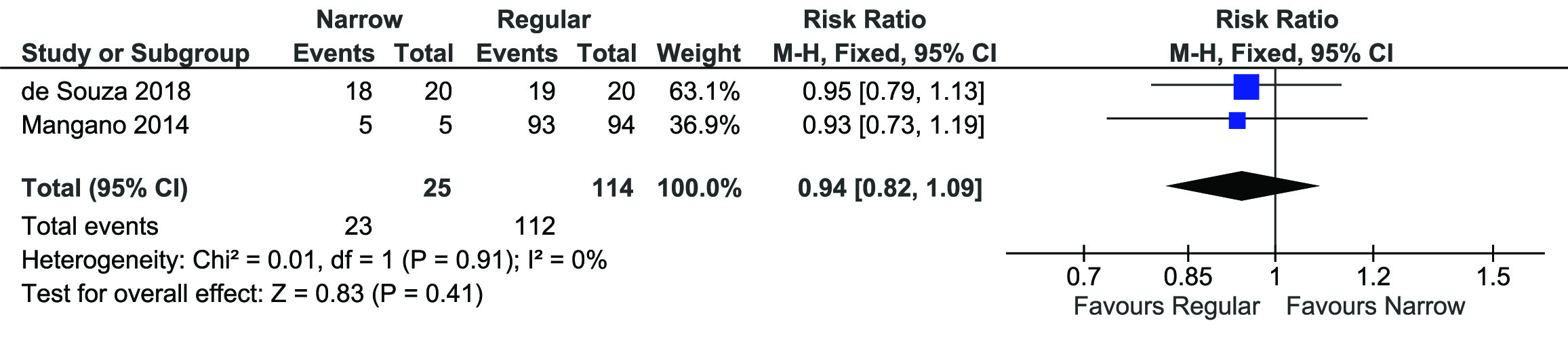

Supplement: Supplementary file 2 — Additional file 2. [file 12903_2023_2962_MOESM2_ESM.jpg]
